# Clinical efficacy of enhanced recovery after surgery in percutaneous nephrolithotripsy: a randomized controlled trial

**DOI:** 10.1186/s12894-020-00728-w

**Published:** 2020-10-20

**Authors:** Qing Li, Li Wan, Shucheng Liu, Mingyong Li, Libo Chen, Zhengwu Hou, Wang Zhang

**Affiliations:** 1 Department of Urology, The First Affiliated Hospital of University of South China, Hengyang, 421001 China; 2 Department of Urology, The Fifth Affiliated Hospital of Southern Medical University, Guangzhou, 510900 China

**Keywords:** Enhanced recovery after surgery, Perioperative management, Percutaneous nephrolithotripsy, Renal calculi

## Abstract

**Background:**

To evaluate the feasibility, safety, applied value and efficacy of enhanced recovery after surgery (ERAS) for PCNL for the treatment of renal calculi. Although the ERAS is applied for many urological diseases, its application in percutaneous nephrolithotripsy (PCNL) is still limited.

**Methods:**

This was a prospective study of patients admitted to hospital January and December 2018 and who were only diagnosed with renal calculi and excepted for serious or uncontrollable basic diseases and patients with multiple operation history and medication history. Patients were randomized 1:1 to the ERAS and traditional operation groups starting on the day before operation and end on the day of discharge. Each group was 118 cases. The stone clearance rate, visual analogue scale (VAS) pain score, the occurrence of perirenal hematoma and effusion, the incidence of extravasation of urine, the incidence of fever, bleeding and blood transfusion, and postoperative hospital stay were observed.

**Results:**

The stone clearance rates were similar between the two groups (ERAS: 93.2% (109/117) vs. traditional: 89.8% (106/118), *P* = 0.800). The operation time was similar in the two groups (ERAS: 54 ± 12 vs. traditional: 58 ± 11 min, *P* = 0.656). VAS pain score that was 0.79 ± 0.76 in the ERAS group at 4 h after surgery and was significantly lower than 2.79 ± 0.98 in the traditional group (*P* < 0.0001). The total complication rate was 15 cases in the ERAS group and 22 cases in the traditional group (*P* = 0.573). There were no difference in costs (21,348 ± 2404 vs. 21,597 ± 2293 RMB, *P* = 0.529).

**Conclusions:**

ERAS perioperative management in PCNL was feasible, was without additional complications, and had well economic and social benefits. It is worth of clinical promotion and application.

## Background

Renal calculi are one of the most common diseases in urology. The reported lifetime prevalence vary from 1 to 20% [1] and appears to be increasing [2]. A national cross-sectional study suggested that the prevalence of renal calculi in Chinese adults was 6.4% [3]. Percutaneous nephrolithotripsy (PCNL) is one of important surgery methods to treat renal calculi [4].

Enhanced recovery after surgery (ERAS), also called "fast-track surgery" (FTS) can effectively reduce surgical stress and complications, accelerate postoperative rehabilitation, and reduce the physiological, psychological, and economic burden of surgeries [5–7]. ERAS Society in Europe had already popularized the ERAS concept in multiple fields: gastrointestinal surgery, hepatobiliary surgery, cardiothoracic surgery, and many others [8, 9].

Aim of our study was to examine the safety, feasibility, economic value, and applied value of ERAS perioperative concept in PCNL for the treatment of renal calculi. The urological ERAS in China is still at the trial stage, without clear guidance or support of high-quality evidence-based medicine, while ERAS has been successfully applied for a variety of urological diseases in western countries [10, 11]. Application of ERAS perioperative management in PCNL could help accelerate patients’ postoperative rehabilitation and reduce postoperative physiologic and psychological stress response. Considering the economic and social benefits, such a strategy is worthy of clinical trial and application. Therefore, The results should help popularize the application of ERAS in PCNL, which is of clinical importance because of the important numbers of patients undergoing PCNL each year.

## Methods

### Study design and patients

This was a prospective study of patients (16–85 years of age) admitted to the hospital between January and December 2018 and who were preoperatively diagnosed with renal calculi by abdominal computer tomography (CT) [12]. The study was approved by the ethics committee of the First Affiliated Hospital of University of South China. All patients provided written informed consent prior to any study procedure. For a type 1 error of 0.05 and a power of 0.8, the number of participants needed for each group was 111. Considering that some patients may quit this study, we aimed to recruit 120 participants per group (240 in total).

The indications and contraindications of PCNL were as published [12]. The inclusion criteria were: (1) diagnosed with renal calculus by abdominal CT; (2) scheduled to undergo PCNL; (3) ASA grade I or II; (4) 16–85 years of age; (5) no uncontrolled renal insufficiency (CDK ≤ 3), uncontrolled diabetes (postprandial blood glucose ≤ 11.1 mmol/L), hypertension (systolic blood pressure ≤ 140 mmHg and diastolic blood pressure ≤ 100 mmHg), cardiac insufficiency (NYHA ≤ 3), and chronic obstructive pulmonary disease (mMRC ≤ 3).

The exclusion criteria were: (1) massive intrarenal calculi (stone length > 4 cm); (2) bilateral renal multiple calculi; (3) severe upper urinary tract malformations such as horseshoe kidney malformation, ureteropelvic stenosis (UPJO), giant ureter disease (POM), and other combined calculi that requires complicated surgery, longer operative time, multiple surgeries, or other factors affecting efficacy evaluation; (4) disease considered to affect the process of surgery, postoperative rehabilitation, prognosis and cost; (5) patient with septic shock; (6) requirement for emergency surgery like catheterization or fistula; (7) ipsilateral upper urinary tract surgery history, (8) active severe infection,(as PCT > 0.5, leukocyte + in urine and other patients with UROGENOUS sepsis diagnosis). (9) Patients taking anticoagulant drugs such as aspirin and warfarin in recent 1–2 weeks. The surgery could be performed only when the coagulation function was with normal results.

PCNL was performed by a single surgeon under general anaesthesia in both group. After induction of anaesthesia, with the patient in lithotomy, a 4-F urethral catheter was inserted into the ureter via cystoscopy. Then the patient was repositioned to prone. Then, an 18-G access needle was placed into the preferred calyx under ultrasound guidance. A tiny incision was made in the skin and fascia, and then the 18-F fascial dilator was used to dilate the nephrostomy tract to pass the 18-F semi-rigid plastic sheath. Then, a 9.8-F, 33-cm semi-rigid ureteroscope (Richard Wolf Medical Instruments, Ver- non Hills, IL, USA) was introduced to the sheath. The renal stones were broken into pieces using Holmium laser. Fragmented stones too large for spontaneous passage from the ureter were removed using a grasper. Then a ureter stent was inserted into ureter anterogradely after ureteric catheter removed. Finally, A 16-F nephrostomy tube was placed into calyx through the sheath, then the sheath was removed.

### Randomization and blind method

The patients were randomized 1:1 to the ERAS and traditional operation groups using sequential sealed opaque envelopes prepared by a third party biostatistician using a random number table and used double blind method.

### Preoperative preparation in all patients

Routine preoperative preparation included blood routine, midcourse urine routine, urine bacterial culture and drug sensitivity test, liver and kidney function, blood coagulation function, intravenous pyelography (IVP), and urinary CT to determine location, size, and number of the stone, split renal function, and anatomical structure. Sensitive antibiotics were used empirically [12] if there were symptoms of urinary tract infection before surgery.

### Preoperative preparation in the ERAS group

In the ERAS group, the patients were let to initially accept and cooperate. Education about the ERAS concept was carried out. The patients received detailed preoperative conversation, including PCNL advantages and disadvantages, advantages of compound anesthesia, rough expenses, perioperative complications (such as bleeding, infection, residue, stones recurrence, early manifestations, prognosis, and treatment measures), and the importance of cooperation. Preoperative nervous hypertension was managed by sublingual administration of 0.5 mg of nitroglycerin (or 50 mg of isosorbide mononitrate sustained-release caspsule) and comforting to reduce preoperative anxiety and mental stress. Blood glucose was monitored and controlled to < 6 mmol/L to prevent stress hyperglycemia and insulin resistance.

No routine preoperative bowel preparation was performed, except for patients with long-term constipation and dry stool hardening, who received cleaning enema. Otherwise, all patients were fasted from solid food for 8 h and received 250 mL of 5% glucose solution 2 h before surgery (diabetic patients received xylitol instead of glucose).

For multimodal analgesia, preemptive analgesia was used 30 min before surgery using parecoxib 40 mg or flurbiprofen 50 mg infusion, and dexamethasone 10 mg static infusion for reducing postoperative wound inflammation, improving the antiemetic effect of 5-HT_3_ receptor blocker, and reducing postoperative insulin resistance. At 30 min before surgery, third generation cephalosporin was given as prophylaxis.

### Preoperative preparation in the ERAS group

The patients were informed of the surgical risks and postoperative complications, and their understanding was obtained. Traditional preoperative intestinal preparation was carried out. The patients fasted overnight and were fasted from all liquids at 4 o'clock in the morning. Parenteral nutrition (glucose and sodium chloride 500 mL iv, vitamins, and potassium chloride) was given to the patients scheduled for surgery late in the day. Third-generation cephalosporin was given as prophylaxis 30 min before surgery.

### Intraoperative management in the ERAS group

The patient underwent PCNL under general anesthesia using compound general intravenous anesthesia and epidural anesthesia or paravertebral nerve block anesthesia (ultrasound-guided). Intraoperative temperature was routinely maintained over 36 °C. At the end of the operation, 5 mg of silansetron (or 50 mL of granisetron sodium chloride) were given intravenously to prevent vomiting, and 2 mL of flumacinib were given intravenously to reverse anesthesia.

### Intraoperative management in the traditional group

The patients received traditional general anesthesia. At the end of the operation, 2 mL of flumacinib were given to reverse anesthesia.

### Postoperative management in the ERAS group

Discharge procedures were completed 1 day after the removal of the nephrostomy tube and when conforming to the discharge criteria. Postoperative visual analog scale (VAS) was pain was used. When coming back to the ward, intramuscular injection of parecoxib 40 mg (or flurbiprofen 50 mg intravenous drip) was done to stop the pain and 15 mL of saline was given orally. One hour later (based on the half-life of 2.5 h for sufentanil), the VAS score was determined again. If VAS was > 4, 2 mL of diclofanac sodium and lidocaine hydrochloride were injected to relieve pain. On the 1st day after surgery, acesodyne was changed to ibuprofen (15 mL orally qd) or celecoxib (200 mg orally qd). At 4 h after surgery, 250 mL of 5% glucose (xylitol for diabetic patients) were given orally, and 5 mg of methoxyclozapine were intramuscularly injected to avoid vomiting. Another dose of 5 mg could be given if nausea still occurred. If there was no serious discomfort, the patients returned to liquid diet 6 h after surgery, and to normal diet the next day. For elderly patients, 0.7 g of Malen capsule orally (bid) or 20 mL of Simo (If it can’t be purchased, Macrogol 4000 powder can be used instead) decoction orally (tid) were given when resuming diet to avoid constipation. Postoperative intravenous fluid volume was reduced beyond 1500 mL. If there was no obvious fever, the nephrostomy tube was clamped 2 or 3 days after surgery. After CT, the catheter was removed. Suitable activity on the bed was encouraged on the first day postoperatively. A mild activity out bed was encouraged on the 3 or 4 days after surgery (Additional file 1: Supplementary Table S1).

### Postoperative management in the traditional group

If the patient complained of unbearable pain, an intramuscular injection of 2 mL of diclofanac sodium and lidocaine hydrochloride was given to relieve pain. Hyperemesis was broken by an intramuscular injection of 5 mg of methoxyclozapine, and 50 mL of intravenous granisetron were added if there were no improvement. The patient was allowed to drink postoperatively and received liquid diet after the first defecation. Absolute bed rest was prescribed for 3–4 days after surgery. CT was performed on the 3rd or 4th days after surgery. The nephrostomy tube was clipped and bed exercises were encouraged. The urethral catheter was removed 4 or 5 days after surgery, and 5–6 days for nephrostomy tube (Additional file 1: Supplementary Table S1).

### Observational indicators

General information, such as sex, age, stone size, stone location, diagnosis, and comorbidities were assessed at baseline. Stone clearance rate [13] and operation time were assessed immediately after operation. Complications, RIRS rate, costs, renal hemorrhage (defined as hematuresis and hemoglobin decrease [14]), indwelling time (nephrostomy tube and catheter), and length of stay were assessed just before discharge. Secondary hemorrhage, urinary fistula, perirenal hematoma, recurrent calculus, and acute renal dysfunction were assessed at 1 month after discharge by follow-up.

### Endpoints

The primary endpoints were VAS, blood loss, extubation time, length of hospital stays, costs, and 30-day follow-up that including secondary hemorrhage, urinary fistula, perirenal hematoma, stone recurrence, acute renal insufficiency and other adverse complications. The secondary endpoints were operation time, stone clearance rate, incidence of RIRS, hemorrhage, and blood transfusion.

### Discharge criteria

The patients we discharge one day after removing nephrostomy tube, without fever, no chills, no septic shock, no active bleeding, imaging examination showed that D-J tube location was reasonable, defecated, no nausea, no vomiting, no abdominal pain, no ileus after eating, fistula not obviously bleeding and leakage, no obvious hematuria, and ambulation [15]. There was no stone residue (residual stone < 5 mm) [9] or residual stone (residual stone > 5 mm) that did not cause urinary tract obstruction without will for a second operation.

### Statistical analysis

R 3.4.3 (https://www.r-project.org) and R Studio 1.1.385 (https://www.rstudio.com) were used for data analysis, based on a per protocol approach. Continuous data were tested for normal distribution using the Kolmogorov–Smirnov test, are presented as mean ± standard deviation (normal distribution) and median (range) (non-normal distribution), and were analyzed using the Student t test or the Mann–Whitney U test. Categorical data are presented as frequencies and were analyzed using the Pearson chi-square test or the Fisher exact test, as appropriate. *P* values ≤ 0.05 were considered to be statistically significant.

## Results

### Characteristics of the patients

**Fig. 1:**
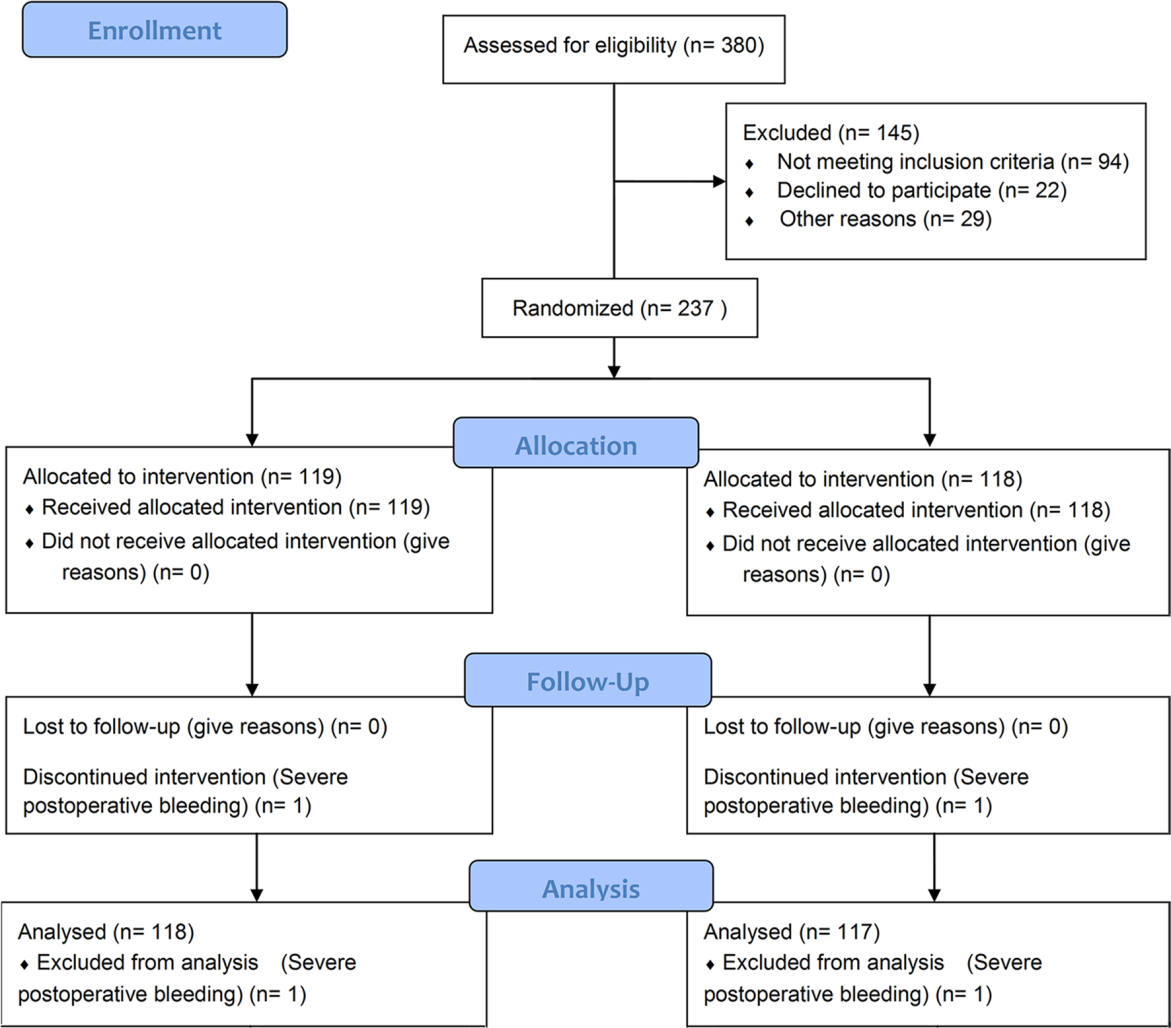
Patient flowchart

Figure 1 presents the patient flowchart. Three hundred patients were assessed for eligibility and 65 were excluded; 235 patients were randomized to the ERAS group (n = 117) and to the traditional group (n = 118).

The average age of the patients in the ERAS group was 51 ± 11 years, and the average stone size was 21.0 ± 9.4 mm. The average age of the patients in the traditional operation group was 54 ± 12 years old, and the average stone size was 19.2 ± 8.2 mm. There were no significant differences between the two groups in sex, age, stone size, and surgical method (all *P* > 0.05) (Table 1).

**Table 1:** Baseline characteristics of the patients

Group	ERAS (117)	Traditional (118)	Χ^2^/t	*P*
Male/female	72/45	74/44	0.0026	0.9594
Age (years)	51 ± 11	54 ± 12	− 1.4834	0.1393
Stone size (mm)	21.03 ± 9.43	19.19 ± 8.16	1.6653	0.0973
Stone location (ureteropelvic/renal)	51/66	53,564	0.0054	0.9416
Hb	129 ± 18	131 ± 17	0.3942	0.6938

### Surgical outcomes

Table 2 presents the surgical outcomes of the patients. None of the patients had failed puncture and had a conversion to open surgery. The ERAS group had a stone clearance rate of 93.2% (109/117), which is not significantly different from 89.8% in the traditional group (106/118) (*P* = 0.800). The average operation time in the ERAS group was 54 ± 12 min, which was not significantly different from 58 ± 11 min in the traditional group (*P* = 0.656). The immediate VAS pain score in the ERAS group was 0.01 ± 0.09, and was 0.39 ± 0.49 in the traditional group (*P* = 0.176). VAS pain score that was 0.79 ± 0.76 in the ERAS group at 4 h after surgery and was significantly lower than 2.79 ± 0.98 in the traditional group (*P* < 0.0001).

**Table 2:** Surgical characteristics

Observation item	ERAS group	Traditional operation group	*P*
Operation time (min)	54.2 ± 12	58 ± 11	0.6561
Decrease of hemoglobin (g/L)	4.79 ± 5.63	8.34 ± 7.23	< 0.0001
Stone clearance rate immediately after surgery	93.2%	89.8%	0.7997
Postoperative VAS	0.01 ± 0.09	0.39 ± 0.49	0.176*
VAS 4 h after surgery	0.79 ± 0.76	2.79 ± 0.98	< 0.0001*
Renal bleeding before discharge	2	10	0.0403
Fever > 38 °C	5	6	> 0.999
Postoperative hospitalization (days)	4.6 ± 1.0	6.2 ± 1.1	< 0.0001
Indwelling fistula time (days)	3.6 ± 1.0	5.2 ± 1.1	< 0.0001
Indwelling catheter time (days)	2.6 ± 1.0	4.2 ± 1.1	< 0.0001
Hospitalization costs (RMB)	21,348 ± 2404	21,597 ± 2293	0.5289
Thrombosis of renal artery	0	0	> 0.999
Postoperative shock, death, organ damage and MODS	0	0	> 0.999

^*^Mann–Whitney U test

### Complications

Table 3 presents the complications. The decrease in hemoglobin was 4.79 ± 5.63 g/L in the ERAS group, which was significantly lower than 8.34 ± 7.23 g/L in the traditional group (*P* < 0.01). In the ERAS group, the urine color was slightly red on postoperative day 1, and normalized with symptomatic treatment including hemostasis, with significantly smaller number of patients with slightly red urine before discharge than in the traditional surgery group (2 vs. 10, *P* = 0.04). None of the patients were observed with impairment of liver, gallbladder, spleen, pancreas, intestines, or other organs. One case of postoperative renal hemorrhage occurred in each of the two groups, and they were relieved after transfusion and selective renal artery embolization (*P* > 0.999).

**Table 3:** Complications

Items	ERAS (n = 117)	Traditional (n = 118)	*P*
Hematuresis	2	10	0.040
SIRS	5	6	> 0.999
Total complications	15	22	0.573
Renal arterial embolization	1	1	> 0.999

No obvious perirenal hematoma and urinary extravasation were found at 1-month follow-up. There were no significant difference in postoperative SIRS risk between the ERAS and traditional groups (*P* > 0.999). The total complication rate was 15 cases in the ERAS group and 22 cases in the traditional group (*P* = 0.573). Therefore, ERAS did not increase the incidence of postoperative complications.

## Discussion

Surgery is a process of treating and repairing lesions and injury but it also involves significant trauma [9]. The stress response caused by the trauma can directly affect the convalescence [16, 17]. There are many causes of stress during the entire process of an operation, such as mental stress before the operation, hunger, thirst, additional procedures, complications, nausea, etc.

Preoperative education is a key factor for ERAS. Effective preoperative education can help patients better understanding the treatment process, reducing psychological pressure and improving patients’ compliance. In advance of analgesia, using anti-inflammatory drugs and long-term fasting was traditionally considered to significantly reduce nausea, vomiting, and aspiration during anesthesia, but this approach increases the burden on the patients. In the ERAS group, shorter preoperative fasting and the preoperative oral administration of sugary liquid can avoid the loss of body fluid and then prevent hypotension and electrolyte disturbance. No intraoperative aspiration occurred in the patients and the incidence of postoperative nausea and vomiting was lower than that in the traditional group, as supported by a previous study [18].

The traditional approach to surgery believes that nephrostomy tube indwelling after PCNL can play a role in pressing the puncture channel, strengthening hemostasis, strengthening drainage, reducing urine extravasation, and reducing the risk of infection. The patients are also more amenable to a second stage surgery in case of residual stones [19]. Nevertheless, the indwelling nephrostomy tube will increase patient discomfort, with breathing-related pain, hindering early exercise, and increasing the dosage of painkillers, postoperative hospitalization time, and costs [20]. Therefore, some authors believe that early removing or even no nephrostomy tube will not increase the risk of bleeding, infection, and urine extravasation [21]. In the present study, the decrease in postoperative hemoglobin in the ERAS group was significantly lower than in traditional group. In addition, the frequency of hematuria at discharge was also lower for the ERAS group. Mild hematuria was managed using vitamin K_1_ 30 mg and phenol-sulfoethylamine 1.0 g intravenously, rehydration, and diuresis [22]. It may be hypothesized that the main cause of postoperative bleeding is iatrogenic [23] and contact friction between the tube and the renal pelvis mucosa. ERAS can reduce the indwelling fistula time, lessening pain, and increasing surgery acceptability and compliance with the surgeons’ orders, reducing the adverse reactions [7]. Although it cannot reduce the inherent risk of renal bleeding after PCNL at the surgical technique level, it has meaningful effect on reducing the inflammatory response, traumatic stress, fistula friction of oozing blood, pain, and discomfort. Preemptive analgesia and compound anesthesia can effectively reduce the intraoperative anesthesia load and resuscitation time, which are safe and effective during anesthesia resuscitation. Postoperative active analgesia can alleviate pain, so that patients would not refuse to resume exercise because of pain, and also reduce the adverse reactions of the removal of nephrostomy tube and urethral catheter.

It is well known that deficient nutrition is detrimental to postoperative recovery [24]. After major surgery, it may increase hospitalization time and costs [25]. Preoperative nutritional deficiency is an important reason of postoperative metabolic stress, especially postoperative insulin resistance, and it usually appears a few minutes after surgery and continues for weeks or even months. This will lead to weakness and increase mortality by 43% in severe patients and the incidence of postoperative complications such as sepsis and kidney failure by 40–50% [26, 27]. Therefore, shortening perioperative fasting, feeding oral energy mixture preoperatively, reducing the liquid load, and controlling blood glucose during hospitalization are conducive to reducing stress and accelerating recovery, significantly improving immune function, nutritional status, and organ function [5, 6]. In the present study, there were no differences in costs between the two groups, which might be because of using some specific drugs during ERAS perioperative management, and because the postoperative hospitalization time was shorter 1.6 ± 0.1 days.

Although the ERAS had been wildly developed and applied in urology, there are several limitations to the present study. First, the application of ERAS in urology, especially for minimally invasive urology surgeries such as PCNL, is still relatively limited. The sample size was limited and from a single center, and the follow-up was short, leading to bias. Furthermore, the lack of quality of evidence-based medical evidence prevented a formal sample size analysis [28]. Conclusions for safety, efficacy, economy, and feasibility still need to be confirmed and supported by large-scale randomized controlled studies and long-term follow-up. Second, the implementation of ERAS could not be performed by a single clinician. Its success requires the cooperation and support of physicians, surgeons, nurses, and anesthesiologists [29]. Therefore, there are many possible sources of variability and bias. Nevertheless, with the joint efforts of various professional medical staff, ERAS will achieve greater development and benefit more patients.

## Conclusions

In conclusion, on the basis of the strict controlled surgical indications and skillful operation, the application of the perioperative ERAS concept in PCNL accelerates rehabilitation and effectively reduces the stress response, and it is a safe and feasible management strategy for PCNL. Furthermore, ERAS has advantages of relieving postoperative pain, shortening hospitalization time and cost, accelerating bed turnover, and improving medical experience, which have socioeconomic value. Therefore, ERAS for PCNL is worth promoting in clinical practice.

## Supplementary information

**Additional file 1:** **Supplementary Table S1.** Comparison of the ERAS and standard managements.

## Data Availability

The datasets used and/or analysed during the current study are available from the corresponding author on reasonable request.

## Abbreviations

ERAS: enhanced recovery after surgery
PCNL: percutaneous nephrolithotripsy
VAS: visual analogue scale
FTS: fast-track surgery
PCT: procalcitonin
CT: computer tomography
UPJO: ureteropelvic junction obstruction
IVP: intravenous pyelography
VAS: visual analog scale
