# Supplementary material for: Clinical efficacy of enhanced recovery after surgery in percutaneous nephrolithotripsy: a randomized controlled trial
Source: BMC Urol. 2020 Oct 20;20:162. doi: 10.1186/s12894-020-00728-w (PMC7574411; doi:10.1186/s12894-020-00728-w)
Supplement: Supplementary file 1 — Additional file 1: Supplementary Table S1. Comparison of the ERAS and standard managements. [file 12894_2020_728_MOESM1_ESM.docx]

**Supplementary Table S1.** Comparison of the ERAS and standard managements

| Grouping | Preoperation | Intraoperation | Postoperation |
| --- | --- | --- | --- |
| ERAS | 1. Preoperative education  2. Reduce stress  3.Reduce insulin resistance  4. Liquid diet and sugar water  5. Short-term fasting  6. Preemptive analgesia  7. Antibiotic prophylaxis  8. Thromboprophylaxis | 1. Short-term analgesia  2. Reduce liquid load  3. General combined local anesthesia  4. Keep temperature (heating blanket and heating salinger)  5. Routinely prevent vomiting | 1. General and epidural anesthesia  2. No/short-term indwelling renal fistula  3. Facilitate gastrointestinal motility  4. Reduce intravenous fluid volume  5. Positive anti-nausea  6. Early remove catheter  7. NSAIDs orally  8. Early recovery activity  9. VAS score |
| Standard | 1. Preoperative conversation  2. Long-term fasting  3. Antibiotic prophylaxis | 1. General anesthesia  2. No routine prevention of vomiting | 1. Pain relief when necessary  2. long-term indwelling renal fistula  3. fasting until venting  4. stay in bed until extracting fistula  5. VAS score |
